# Mechanical quantitative sensory testing in cavalier King Charles spaniels with and without syringomyelia

**DOI:** 10.1186/s12917-020-02313-7

**Published:** 2020-03-20

**Authors:** Ashley C. Hechler, Eric T. Hostnik, Laurie B. Cook, Lynette K. Cole, Sarah A. Moore

**Affiliations:** The Ohio State University Veterinary Medical Center, 601 Vernon L. Tharp Street, Columbus, OH 43221 USA

**Keywords:** Chiari-like malformation, Syringomyelia, Neuropathic pain, Quantitative sensory testing, von Frey aesthesiometry, Cavalier King Charles spaniel

## Abstract

**Background:**

Syringomyelia (SM) is a debilitating condition in the cavalier King Charles spaniel (CKCS) that results in neuropathic pain and diminished quality of life. Von Frey aesthesiometry (VFA) is a method of mechanical quantitative sensory testing that provides an objective sensory threshold (ST) value and can be used to quantify neuropathic pain (NP) and monitor response to therapy. The utility of VFA has been previously established in client-owned dogs with acute spinal cord injury but the technique has not been evaluated in dogs with SM. The goal of this study was to evaluate ST, as determined by VFA, in dogs with and without SM, to assess the utility of VFA in quantifying NP in SM-affected dogs. We hypothesized the SM-affected CKCS would have lower ST values, consistent with hyperesthesia, when compared to control CKCS. Additionally, we hypothesized that ST values in SM-affected dogs would be inversely correlated with syrinx size on MRI and with owner-derived clinical sign scores.

**Results:**

ST values for the thoracic and pelvic limbs differed significantly between the SM-affected and control CKCS (*p* = 0.027; *p* = 0.0396 respectively). Median ST value (range) for the thoracic limbs was 184.1 g (120.9–552) for control dogs, and 139.9 g (52.6–250.9) for SM-affected dogs. The median ST value (range) for the pelvic limbs was 164.9 g (100.8–260.3) in control dogs and 129.8 g (57.95–168.4) in SM-affected dogs. The ST values in SM-affected dogs did not correlate with syrinx height on MRI (*r* = 0.314; *p* = 0.137). Owner-reported clinical sign scores showed an inverse correlation with pelvic limb ST values, where dogs with lower ST values (hyperesthesia) were reported by their owners to display more frequent and severe clinical signs (*r* = − 0.657; *p* = 0.022).

**Conclusion:**

ST values were lower in SM-affected CKCS compared to control dogs, suggesting the presence of neuropathic pain. Dogs with lower ST pelvic limb values were perceived by their owners to have more severe clinical signs classically associated with SM. Our results suggest that VFA might offer quantitative assessment of neuropathic pain in SM-affected dogs and could be useful for monitoring response to therapy in future clinical studies.

## Background

Syringomyelia (SM) is an acquired neurologic disorder that can result in debilitating pain in the cavalier King Charles spaniel (CKCS) breed. SM is the development of fluid-filled cavitations in the cervical, thoracic, and occasionally lumbar spinal cord [1, 2], most often affecting the dorsal horn, resulting in presumptive abnormalities of sensory processing due to disruption of the normal pathways. In CKCS, SM results secondary to a malformation of the skull termed Chiari-like malformation(CM), both of which are diagnosed using magnetic resonance imaging (MRI). Clinical signs of SM include an array of behaviors such as compulsive scratching of the neck and flank, unprovoked vocalizations, and apparent sensitivity to light touch [1, 3–8]. While these clinical signs have long been suspected to represent behavioral manifestations of neuropathic pain, studies using quantitative measures of sensation to document a neuropathic pain state in SM-affected dogs are limited [9].

In addition to being a disease of substantial importance within the CKCS breed, SM-affected dogs may represent an important clinical animal model through which to study new interventions aimed at treating neuropathic pain (NP). A canine clinical model of neuropathic pain would complement existing experimental laboratory animal models by offering the ability to study a naturally occurring disease, in a clinical setting, with canine patients who are diagnosed and managed in a similar fashion to people with the same disease. Of equal importance, developing objective measures of NP in dogs with SM might allow more targeted studies of medical and surgical interventions aimed at improving outcome in affected canine patients and their human counterparts. Von Frey aesthesiometry (VFA) is a non-invasive, mechanical quantitative sensory test that delivers a known amount of punctate pressure to a body region until a behavioral manifestation of pain is elicited. The value obtained is termed the sensory threshold and alterations in this number can detect the presence of hyperesthesia and hypoesthesia [10, 11]. Hyperesthesia, encompassing both allodynia and hyperalgesia, is a common manifestation of neuropathic pain in people (14). Von Frey Aesthesiometry is used in people and laboratory rats to measure punctate hyperesthesia mediated through Aδ-fibers as a representative value for the severity of neuropathic pain resulting from central sensitization [10–12].

The goal of the present study was to conduct an investigation of mechanical quantitative sensory testing (QST), a technique for measuring neuropathic pain, using VFA in CKCS with and without SM. The primary aim was to identify the presence of hyperesthesia in SM-affected dogs manifested by lower sensory threshold (ST) values compared to control dogs. A secondary aim was to evaluate the association between VFA-acquired ST values and severity of SM as documented on magnetic resonance imaging (MRI), and via a previously validated owner-derived score of clinical signs using a questionnaire specifically designed for CKCS with SM [6]. We hypothesized that dogs with SM would have significantly lower ST values than dogs without SM, consistent with hyperesthesia. We also hypothesized that ST values in SM-affected dogs would be inversely correlated with MRI severity of SM and with severity of owner-reported clinical signs of SM.

## Results

### Case selection

Nineteen SM-affected and 10 control CKCS were prospectively enrolled from a population of 46. Demographic factors (age, sex) for all dogs included in the present study are summarized in Table 1 and enrollment process is summarized in a CONSORT flow diagram (Supplementary Material). Control dogs ranged from 1 to 2 years of age (median 1.5 years) and SM-affected dogs ranged from 1 to 8 years of age (median 4 years). SM-affected dogs were significantly older than control dogs (*p* = 0.037), but the sex did not differ significantly between groups.

**Table 1:** Demographic factors (age, sex) for 29 CKCS with and without syringomyelia (SM)

Patient	Age (years)	Sex	CM Grade	SM Grade
*Control*				
1	1.5	MN	1	0
2	1	FI	2	0
3	1.5	MN	1	0
4	1.5	FS	1	0
5	1.5	MI	1	0
6	1	MI	2	0
7	1.5	MN	2	0
8	1	FI	1	0
9	2	MN	1	0
10	1	MN	1	0
*SM-Affected*				
1	5	MN	2	1
2	8	FS	2	2
3	7	MN	2	2
4	5	FS	2	2
5	8	FS	2	2
6	6	FS	2	2
7	6	MN	1	2
8	6	MN	1	2
9	4	MN	2	2
10	3	MN	1	1
11	1.5	FI	1	1
12	1.5	FI	1	1
13	1.5	MI	2	2
14	1.5	FI	1	2
15	1	FS	2	2
16	5	FS	2	2
17	3	FS	2	2
18	1	FS	2	1
19	1	MI	2	2

*CM* Chiari-like malformation, *SM* Syringomyelia, *MI*, Intact male, *MN* Neutered male, *FI* Intact female, *FS* Spayed female

### Magnetic resonance imaging findings

Individual MRI findings for control and SM-affected dogs are summarized in Table 2. Ten dogs had no evidence of SM and served as control dogs, while 19 dogs had evidence of SM on MRI and were termed “SM-affected”. Using the BVA SM grading scale [13] to further delineate the severity of syringes on MRI, a diagnosis of grade 1 SM (SM1) was present in 5 dogs and grade 2 SM (SM2) in 14 dogs. The syrinx height ranged from 0.87 to 1.7 mm (median 1.2 mm) in SM1 dogs, and 2.2 to 9.26 mm (median 3.6 mm) in SM2 dogs.

**Table 2:** MRI findings for 10 control and 19 syringomyelia (SM)-affected CKCS

Patient	PSOM	SM Height (mm)
*Control*		
1	N	–
2	N	–
3	N	–
4	L	–
5	B	–
6	N	–
7	N	–
8	N	–
9	L	–
10	N	–
*SM-Affected*		
1	N	1.119
2	N	6.028
3	L	4.585
4	R	4.94
5	R	9.26
6	N	3.537
7	N	3.059
8	N	2.2125
9	N	2.438
10	R	0.875
11	N	0.933
12	B	1.422
13	N	2.755
14	R	2.689
15	N	3.748
16	B	3.2
17	N	4.212
18	R	1.726
19	R	4.668

*B* Bilateral, *L* Left, *N* Normal, *PSOM* Primary secretory otitis media, *R* Right, *SM* Syringomyelia

### Mechanical quantitative sensory testing

**Fig. 1:**
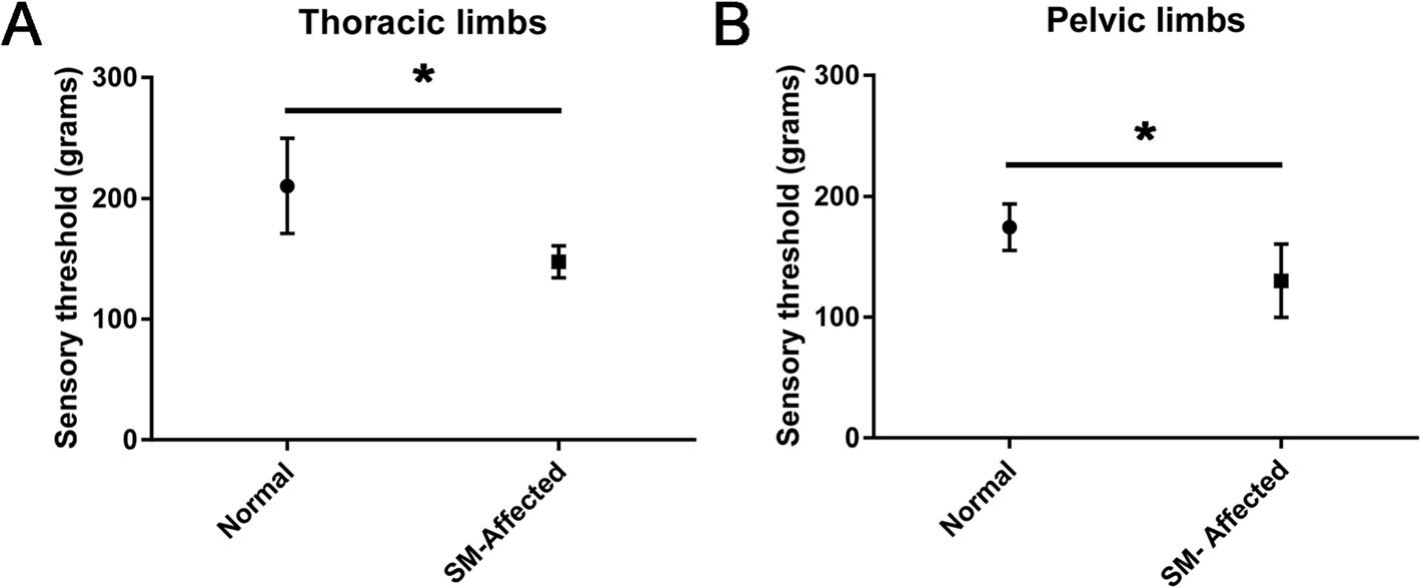
Comparison of median and range sensory threshold (ST) values for thoracic (**a**) and pelvic (**b**) limbs in control and SM-affected CKCS. ST values are significantly lower in the thoracic and pelvic limbs of SM-affected dogs compared to controls, consistent with hyperesthesia and a neuropathic pain phenotype (* denotes *p* < 0.05)

The median sensory threshold (ST) value for the thoracic limbs was 184.1 g (range 120.9–552) in control dogs, and 139.9 g (range 52.6–250.9) in SM-affected dogs. The median ST value (range) for the pelvic limbs was 164.9 g (100.8–260.3) in control dogs and 129.8 g (57.95–168.4) in SM-affected dogs. ST values were significantly lower in the thoracic limbs (*p* = 0.027) and pelvic limbs (*p* = 0.039) of SM-affected dogs when compared to controls (Fig. 1a and b). There were no adverse effects secondary to VFA testing.

### Owner-derived clinical signs scores

Using a previously validated questionnaire to assess owner perception of SM-associated clinical signs [6], SM-affected dogs were assigned a composite score for the severity of clinical signs obtained by averaging the frequency of an assortment of behaviors across several categories. Results for individual animals are summarized in Table 3. This information was available only for SM-affected dogs enrolled in the study on or after January 1, 2017 (*n* = 10). The median owner-derived composite clinical signs score for SM-affected dogs was 1.3 (SM- affected range 0.5–1.8), a clinically normal dog would receive a score of 0.

**Table 3:** Owner-derived clinical sign scores for 10 syringomyelia (SM)-affected CKCS enrolled on or after January 1, 2017. Patient number corresponds to the number listed in Tables 1 and 2

Patient	10	11	12	13	14	15	16	17	18	19
Compulsive scratching	2	4	4	3	3	3	3	2	3	2
Facial rubbing	1	1	3	3	3	3	4	1	2	2
Hypersensitivity to light touch	2	0	4	3	3	2	1	3	2	0
Unexplained yelping	3	0	2	0	2	1	4	0	2	1
Not willing to lift head	2	0	1	1	1	0	1	0	2	1
Not willing to bend neck to eat	2	0	1	0	0	0	1	0	2	0
Weakness or ataxia	2	0	0	0	0	0	0	1	2	0
Strange behaviors	1	0	0	0	0	0	0	0	0	0
Reluctance to defecate	0	0	0	0	0	0	0	0	0	0
Sleep with head elevated	1	0	0	2	2	0	0	0	3	2
**Composite Score**	1.6	0.5	1.5	1.2	1.4	0.9	1.4	0.7	1.8	0.8

0: never; 1: seldom, 2: sometimes; 3: usually; 4: always

#### Sensory threshold values and syrinx size

**Fig. 2:**
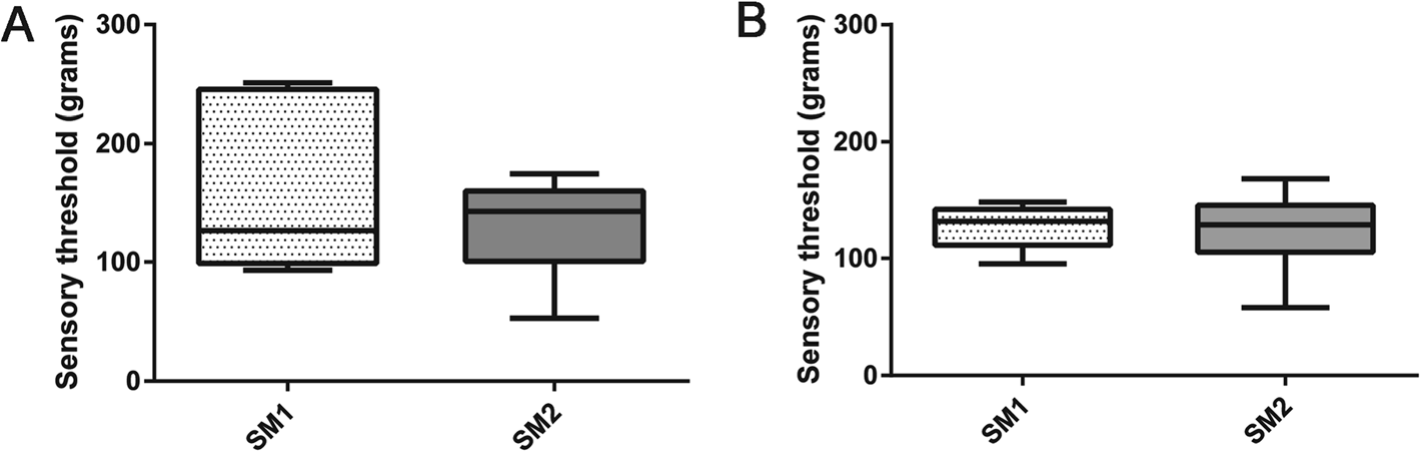
Comparison of median and range sensory threshold (ST) values for thoracic (a) and pelvic (b) limbs in SM1 and SM2-affected CKCS. There is no significant difference in thoracic or pelvic limb ST between SM1 and SM2-affected dogs (*P* > 0.05 in all cases)

When SM-affected dogs were grouped by BVA grade, the median ST value for the thoracic limbs was 104.6 g (range 93–250.9) for the SM1 dogs and 142.8 g (range 49.9–178.9) for the SM2 dogs. In the pelvic limbs, the median ST value was 121.1 g (46–148.3) for SM1 dogs and 114.4 g (58–171.2) for SM2 dogs. There was no significant difference in thoracic or pelvic limb ST values between SM1 and SM2 dogs (*p* = 0.75 and *p* = 0.89, respectively; Fig. 2). The association between ST value and syrinx height, in millimeters, was evaluated for dogs with SM2. There was not a significant correlation between syrinx height and thoracic (*p* = 0.137) or pelvic limb (*p* = 0.324) ST values.

#### ST values and owner-derived SM clinical sign scores

**Fig. 3:**
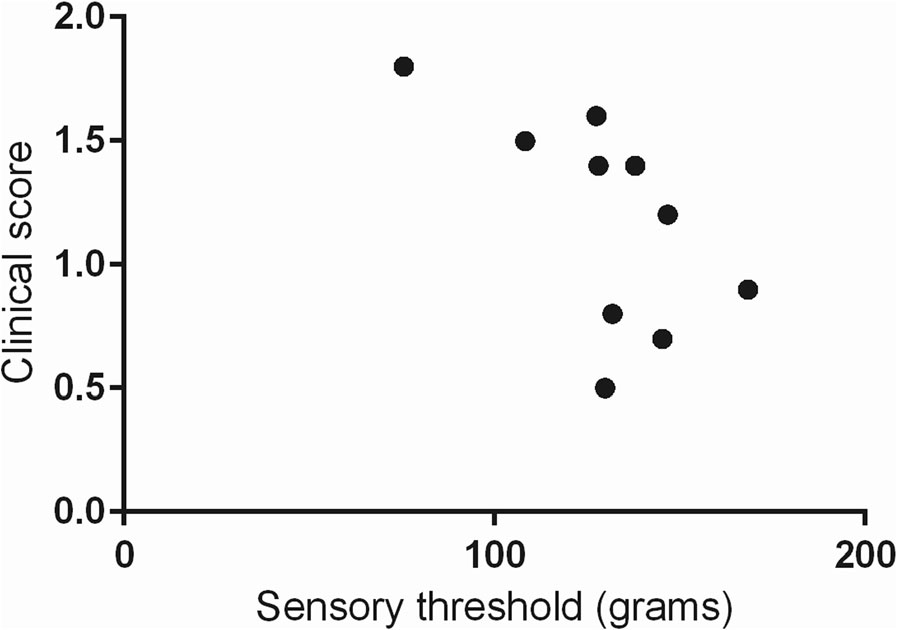
Relationship between sensory threshold (ST) values for pelvic limbs and owner-derived clinical sign scores for SM-affected dogs (*n* = 10). There is a significant inverse correlation between pelvic limb ST values and clinical signs, suggesting that animals with more pronounced hyperesthesia showed more clinical signs of SM at home (*r* = −0.657; *p* = 0.022)

The relationship between owner-derived clinical sign scores and ST values was evaluated for all dogs where this information was available (*n* = 10; Fig. 3). A significant inverse correlation was observed between ST values and owner-reported clinical signs for the pelvic limbs (*r* = − 0.657; *p* = 0.022), but not the thoracic limbs (*r* = − 0.347; *p* = 0.16).

## Discussion

Results of the present study suggest that thoracic and pelvic limb ST values, as measured by VFA, differ between control and SM-affected dogs. SM-affected dogs display lower ST values consistent with a neuropathic pain phenotype. This is not an unexpected finding, as hyperesthesia secondary to central sensitization has long been implicated as the cause of clinical signs in SM-affected dogs; however, this has not previously been quantified. In people, mechanical QST techniques such as VFA are classically used to detect the presence of allodynia. Allodynia can be further classified as dynamic, punctate, or static depending on the type of stimulus that elicits the sensation. Static allodynia, the less commonly recognized form, is typically restricted to the affected area and can be tested using superficial and deep pressure. Punctate allodynia, mediated by Aδ-fibers, most often follows a dermatomal pattern of distribution and is evaluated with von Frey monofilaments or pins [14]. Therefore, ability to identify allodynia relies heavily on the appropriate selection of diagnostic test and body region evaluated. Our study exclusively looked at punctate allodynia by using VFA to test dermatomal patterns commonly affected in SM [15]. The differences in location and prevalence of punctate and static allodynia may explain why our findings differ from another recent study [9] which did not identify a difference in ST values or thermal latencies in CKCS with and without SM. Sparks et al. used hemostatic forceps to apply pressure to the subcutaneous tissues of the neck and thoracic limbs, exclusively evaluating static allodynia [9]. Static allodynia is less frequently reported in people with neuropathic pain, and when reported tend to affect small areas which may be difficult to map in affected dogs [14]. Additionally, several SM-affected dogs in the Sparks et al. study received analgesics immediately prior to the testing procedures. Previous studies have documented increased ST values following analgesic administration [16]. Our study required a one-week washout of all medications prior to enrollment; therefore, eliminating the confounding effect of analgesics on ST values.

The presence of a neuropathic pain phenotype in SM-affected dogs, as documented by VFA, is further supported by the identification of a significant inverse correlation between owner-reported severity of clinical signs and ST values, where SM-affected dogs with lower pelvic limb ST values displayed a higher frequency and severity of clinical behaviors commonly associated with neuropathic pain. Interestingly, this relationship did not hold true for the thoracic limbs. Of consideration here may be the difference between at-level and below-level pain. Immediately following an injury affecting the nerve roots or spinal cord, at-level pain develops in the dermatomes of the affected spinal cord segment which may resolve over time [17]. Below-level pain develops later from central sensitization, affecting dermatomes caudal to the site of injury [18], and has been associated with higher pain scores than at-level neuropathic pain in people [19]. Below-level pain can persist longer and is often more difficult to treat [20], mirroring the clinical course for some SM-affected dogs who can be quite refractory to analgesics. It is possible that many of the pain-related questions, asked of owners to derive a clinical signs score, focus more on manifestations of below-level pain (for example, hypersensitivity to light touch, where some of our dogs scored the highest). Alternatively, our small sample size may have created difficulty in identifying a statistical relationship given the higher variability in the thoracic limb ST dataset for SM-affected dogs. Because only 10 dogs without SM were able to be recruited for the study, it is possible that our lack of ability to establish differences may represent a Type II statistical error and that study in a larger population of animals may be warranted. It is important to note this study only included images of the cervical and cranial thoracic spinal cord, so it is possible a portion of the population had multifocal or holocord syringomyelia which would contribute to the distribution of pain.

An unexpected finding in the present study was the lack of relationship between ST values in SM-affected dogs and syrinx height observed on MRI or difference between SM grades using the BVA scheme. While some studies suggest that syrinx size predicts severity of neuropathic pain-related clinical signs, there is conflicting evidence regarding this relationship in the human and veterinary literature [3, 7, 9, 21, 22]. Recently, the focus in human and veterinary medicine has shifted from assessing the size of the syrinx to evaluating the symmetry, with asymmetrical syringes more commonly associated with neuropathic pain [3, 7, 23, 24]. Alternatively, our study may have been underpowered to detect these differences, given the small group size. Due to the small number of dogs included in this study, we were unable to assess the effect of asymmetry on ST values; however, this represents an important avenue for future studies. An additional consideration in the present study is that most SM-affected dogs were graded SM2, with only five SM1 dogs included. The small number of SM1 dogs may have confounded our ability to identify a statistically significant difference in ST values between mild and more severely SM-affected dogs.

An important limitation in our study, and one that begs discussion for all CM/SM studies in CKCS, is the difficulty in identifying a true “normal” population for comparison within the breed. While the importance of CM as a contributor to pain has been historically minimized in dogs with SM, it has been documented in both people and dogs to cause pain in the absence of SM [9, 25–27], with or without variable contribution from concurrent craniocervical junction anomalies [28–30]. Selection of CKCS without CM or other minor occipital or skull-based malformations proves near impossible due to the ubiquitous nature of these malformations within the breed [7, 31]. Indeed 100% of the control population in the present study had CM apparent on MRI. Therefore, it is impossible to determine from the present study the degree to which CM alone influences ST values; however, its universal presence in both the control and SM-affected groups suggests that SM is likely the driver of significant differences between the two groups. The commonality of primary secretory otitis media (PSOM) [31] in the breed provides another potential confounder, although PSOM-affected dogs do not always display signs indicative of discomfort [32, 33]. In this study, we attempted to exclude patients with PSOM by requiring otoscopic examination by a board-certified veterinary dermatologist showing a normal appearance of the tympanic membrane prior to enrolling. However, the sensitivity of otoscopic examination for a diagnosis of PSOM is low [33] and several SM-affected dogs had evidence of PSOM on MRI. Because normal CKCS used in the present study were recruited from another, unrelated trial, the potential also exists that some degree of selection bias may have been present in the control population that could influence the documented changes between groups.

## Conclusion

VFA is a non-invasive test that can be used to document the presence of neuropathic pain. In the current study, ST values were lower in SM-affected dogs compared to controls, indicating hyperesthesia which is suggestive of a neuropathic pain phenotype. Additionally, ST pelvic limb values were inversely correlated with owner-reported severity of clinical signs suggesting that hyperesthetic dogs displayed more frequent and severe clinical signs of SM. While ST values did not correlate with imaging severity of SM as assessed using syrinx size, additional studies are needed to assess the relationship between other imaging parameters such as asymmetry of syringes and results of mechanical QST. Future studies should focus on the effect of medical and surgical interventions on ST values in SM-affected dogs and should work to define the influence on ST values of other common comorbidities of the head and cervical spine in CKCS.

## Methods

Client-owned CKCS enrolled in the current study were presented to OSU Veterinary Medical Center between June 2010 and June 2018. In total, twenty-nine dogs were enrolled; 10 cavalier King Charles spaniels (CKCS) free of imaging characteristics of SM and 19 CKCS with SM documented on MRI. Sample size determinations were based on pilot data from seven normal and five SM-affected CKCS. The results suggested 15 dogs per group were necessary to detect a 50% difference between normal and SM-affected ST with 90% power and alpha = 0.05. Dogs were included if they were greater than 1 year of age, were cardiovascularly stable for general anesthesia, and were free of significant dermatologic or otologic disease as determined by a board-certified veterinary dermatologist (LKC) on physical and dermatologic examination. Dogs were excluded if the tympanic membrane was bulging on otoscopic examination, if they had any evidence of concurrent neurologic or dermatologic disease on physical examination, or if separation anxiety prevented diagnostic work-up. Dogs less than 2 years of age had a PCV/TS, while the remainder had complete blood counts and biochemistry profiles suggesting general good health. At the completion of the study, all dogs were discharged and returned to their owners.

### Control dogs (*n* = 10)

Ten apparently healthy CKCS were recruited from a population of 29 dogs presenting to The Ohio State University Veterinary Medical Center Dermatology Service (LKC) for an unrelated study. While our sample size calculation originally suggested that at least 15 dogs per group would be required, enrollment was truncated at 10 due to challenges with identifying dogs without SM on MRI and the eventual conclusion of the unrelated study. Dogs were free of significant abnormalities on a complete neurological examination performed by one of the investigators (SAM), and free of dermatologic and otologic disease as assessed by a board-certified veterinary dermatologist (LKC), and had not received any medications in the 7 days prior to study enrollment. All dogs were assigned a CM grade and classified by a single radiologist (ETH) as having no evidence of SM based on MRI of the brain and cervical spine (SM0 using the British Veterinary Association (BVA) scale; Table 4). The radiologist was blinded to the clinical information and VFA results of each patient.

**Table 4:** Chiari malformation (CM) and syringomyelia (SM) severity scoring strategy, as proposed by the British Veterinary Association (BVA) for use in stratifying dogs with CM/SM [13]

Grade	CM
**CM0**	Cerebellum is rounded with CSF between vermis and foramen magnum
**CM1**	Indentation of cerebellum by supraoccipital bone; CSF present between vermis and foramen magnum
**CM2**	Cerebellar vermis is impacted into or through the foramen magnum
**SM0**	No syrinx or central canal dilation
**SM1**	Central canal dilation with a diameter < 2 mm
**SM2**	Central canal dilation > 2 mm, separate syrinx, or pre-syrinx with or without central canal dilation

### Affected dogs (*n* = 19)

SM-affected CKCS were recruited from a population of 46 dogs presenting to the OSU Veterinary Medical Center Dermatology and Neurology services (LKC or ACH). All dogs underwent a complete dermatologic and neurologic examination performed by one of the investigators (SAM, LKC, ACH). A radiologist (ETH), blinded to clinical status and VFA results, assigned a BVA grade for both CM and SM. The dogs were classified as “SM-affected” based on the presence of imaging characteristics consistent with SM (BVA SM grade 1 or 2) on MRI of the brain and cervical spine (Table 4) [13]. Dogs were not administered any medications in the 7 days prior to study enrollment.

### Questionnaire (*n* = 10)

Owners of SM-affected dogs completed a clinical signs questionnaire previously developed as a semi-quantitative measure of quality of life, neurobehavioral changes and owner perceived pain scores in SM-affected CKCS (*n* = 10) [6, 34]. The questionnaire asked owners to assign a severity score for 10 questions addressing clinical signs based on a 5-point Likert-type rating scale (behavior happens 0 = never, 1 = seldom, 2 = sometimes, 3 = usually, 4 = always) [6, 34]. Frequency scores for each clinical behavior were averaged across categories to provide a single clinical sign score for each dog, resulting in a maximum potential score of 5.

### Von Frey Anesthesiometry

Mechanical QST of all four limbs was performed using an electronic von Frey anesthesiometer (VFA- IITC Life Science; Woodlands, CA) in a method previously described by our laboratory [35, 36]. Limb test order was determined at random after dogs were allowed 15 minutes to acclimate to the investigators. Dogs were prevented from visualizing the device during application to ensure behavioral responses were due to tactile stimulation [37]. The minimum force, in grams, required to elicit a behavioral response (attempt to escape, eye movements, lip licking, vocalization) was recorded five times per leg, with a 1-min break between testing to avoid windup, ST decay, and hypersensitization [37–39]. The highest and lowest ST values obtained from each limb were discarded and the middle three values were averaged to produce a mean ST value per limb for each dog. Immediate withdrawal of the limb without pressure or conscious response was determined to be a reflexive movement and was discarded and the stimulus was repeated after 1 minute [13, 37, 40]. All measurements were made by the same investigator (SAM), who was unaware of SM status at the time of examination and was blinded to owner-reported behavior history.

### Magnetic resonance imaging

An MRI of the brain and cervical spine was performed under general anesthesia in all dogs (*n* = 29). All anesthetic protocols were customized for the individual patient by a board-certified veterinary anesthesiologist or anesthesia resident. The protocols included an intravenous pre-medication and maintenance on isoflurane. Dogs were imaged in dorsal recumbency using a 3.0 Tesla Philips Achieva magnet or 3.0 Tesla Philips Ingenia model magnet (Highland Heights, OH 44143). At minimum, T1-weighted (TR = 450–700 milliseconds; TE = 8 milliseconds) and T2-weighted (TR = 3500–5000 milliseconds; TE = 110 milliseconds) images were obtained in the sagittal plane for review, with additional sequences and transverse images obtained on a case-by-case basis as medically indicated and dependent on presence and location of syringes.

Image interpretation was performed by a single board-certified veterinary radiologist (ETH) blinded to patient clinical history and QST results. A commercially available DICOM viewing software program (Horos2k v. 2.0.2, https://www.horosproject.org) was used to measure syrinx height (in millimeters), at the location of maximum apparent height, and to grade the CM in the sagittal plane. CM and SM grades were assigned using the BVA grading scheme depicted in Table 4 [13]. Concurrent presence or absence of imaging characteristics consistent with PSOM were recorded for each dog as normal, unilateral (denoted as left or right based on affected side), or bilateral [41].

### Statistical analysis –

Age and sex were compared between groups using the Wilcoxin rank-sum test. To facilitate statistical comparisons, ST values for the limbs of each individual dog were averaged to produce a single mean ST value for the thoracic limbs and for the pelvic limbs. Summary data for ST in thoracic and pelvic limbs (g), syrinx height (mm), CM and SM grade, and owner-derived clinical sign scores were reported using descriptive statistics. Data were evaluated for normality using the Shapiro-Wilks test and were reported as median (range) because the data were not normally distributed. The Wilcoxon rank-sum test was used to compare mean ST values between control and SM-affected dogs. Relationships between owner-derived composite clinical sign scores, imaging findings, and ST values were evaluated using Spearman correlations. Statistical analyses were performed using GraphPad Prism and *P* < 0.05 was considered significant for all tests.

## Supplementary information

**Additional file 1.** CONSORT flow diagram depicting the enrollment process.

## Data Availability

The datasets used and/or analyzed during the current study are available from the corresponding author on reasonable request.

## Abbreviations

BVA: British veterinary association
CCJ: Craniocervical junction
CKCS: Cavalier King Charles spaniel
CM: Chiari-like malformation
CM1: Grade 1 Chiari-like malformation
CM2: Grade 2 Chiari-like malformation
CSF: Cerebrospinal fluid
MRI: Magnetic resonance imaging
NP: Neuropathic pain
PSOM: Primary secretory otitis media
QST: Quantitative sensory testing
SCI: Spinal cord injury
SM: Syringomyelia
SM0: Grade 0 syringomyelia
SM1: Grade 1 syringomyelia
SM2: Grade 2 syringomyelia
ST: Sensory threshold
VFA: Von frey anesthesiometry
