# Supplementary material for: Mechanical quantitative sensory testing in cavalier King Charles spaniels with and without syringomyelia
Source: BMC Vet Res. 2020 Mar 20;16:94. doi: 10.1186/s12917-020-02313-7 (PMC7085174; doi:10.1186/s12917-020-02313-7)
Supplement: Supplementary file 1 — Additional file 1. CONSORT flow diagram depicting the enrollment process. [file 12917_2020_2313_MOESM1_ESM.doc]

**
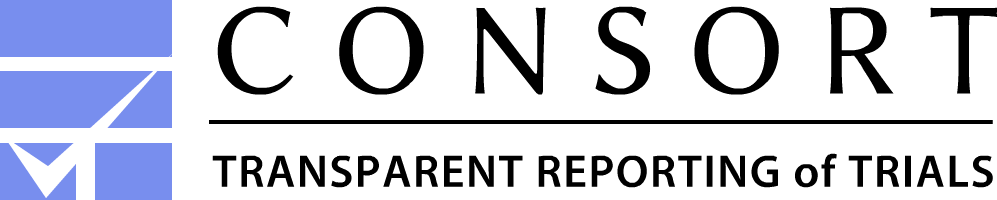
**

**CONSORT 2010 Flow Diagram**

**Enrollment**

Assessed for eligibility through Dermatology service (n= 29 )

Excluded (n= 10)

♦  PSOM (n= 10)

Allocated **SM-affected** (n=19)

♦  Initially presented to dermatology (n= 9)

♦  Initially presented to neurology (n= 10)

Allocated to **Control** (n=10)

Assessed for eligibility through Neurology service (n= 17)

Excluded (n= 9)

♦  PSOM (n= 2)

♦  Otitis Externa (n= 3)

♦  Severe separation anxiety (n=1)

♦  Atopic Dermatitis (n= 2)

♦  Clinical signs too severe to discontinue medications (n= 1)
